# Scientific work and knowledge of scientific methods in local public health authorities (SCOPE) – wish or reality? Results of a semi-standardized cross-sectional survey of local public health authorities in Germany (Part 1)

**DOI:** 10.1371/journal.pone.0345944

**Published:** 2026-04-09

**Authors:** Emily Piontkowski, Anja Herrmann, Hannah Richter, Sofie Wössner, Linda Eichner, Monika A. Rieger, Stefanie Joos, Brigitte Joggerst, Karin Geffert, Rebecca Zöllner, David Häske

**Affiliations:** 1 Centre for Public Health and Health Service Research, University Hospital Tübingen, Tübingen, Germany; 2 Institute of Occupational and Social Medicine and Health Services Research, University Hospital Tübingen, Germany; 3 Institute of General Practice and Interprofessional Care, University Hospital Tübingen, Tübingen, Germany; 4 Gesundheitsamt Karlsruhe, Landratsamt Karlsruhe, Karlsruhe, Germany; 5 Baden-Wuerttemberg Federal State Health Office (LGA), Ministry of Social Affairs, Health and Integration, Stuttgart, Germany; 6 Gesundheitsamt Frankfurt am Main, Frankfurt am Main, Germany; Taipei Medical University, TAIWAN

## Abstract

In Germany, there are efforts to strengthen scientific work in local public health authorities (LHA) to improve efficiency and effectiveness and to ensure quality in the public health service (PHS). This study looks at how LHA staff view their own knowledge of scientific methods, how often they apply these methods, and what scientific structures exist in LHA. An interdisciplinary team created a partially standardized online questionnaire for LHA staff. The survey covered demographics, attitudes towards scientific work in PHS, knowledge of scientific methods, existing research structures in LHA, and participation in research projects. The responses were analysed for both absolute and relative frequencies. The results show that the respondents rarely work scientifically, despite having the necessary knowledge. A comparison of the departments within LHA shows that staff from the field of health reporting gave the highest overall figures for knowledge and frequency of use. Almost three quarters of respondents had not been involved in scientific publications in the last five years. More than half of the respondents do not have access to scientific software or do not use it. Most respondents indicated a need for further training or counselling in scientific methods. These findings indicate that there is a discrepancy between knowledge and practical application of scientific methods and suggest that limited use of scientific methods in LHA is less a matter of motivation than of insufficient structural support. Targeted measures, such as the provision of suitable software or the further development of training and counselling programs could help strengthen, scientific work in LHA and thus improve quality and strengthen the PHS. Addressing barriers such as the insufficient legal and organizational anchoring, limited structural and human resources, and the lack of leadership culture that prioritizes scientific work, may help support evidence-based public health and informed decision-making.

## Background

The public health service (PHS) is responsible for the monitoring of population health, the protection of public health, the promotion of health and the prevention of diseases. In addition to these core functions, the PHS is also tasked with ensuring health equity. In 2012, the World Health Organization (WHO) published the “Essential Public Health Operations” (EPHO) to strengthen PHS in Europe, emphasizing research as a crucial component of policy and practice [1]. The “Public Health Competency Framework”, published in 2014, underscores the need for research and analysis as fundamental competencies in health emergencies [2,3].

In Germany, the PHS is organised on a federal, state and local level. Next to the authority at the federal level, there are 16 authorities at the state level (for each federal state) and subsequently authorities at the local level, the local public health authorities (LHA) [4]. A comparison of the structure of PHS across several countries reveal a tendency towards centralisation. For instance, the “UK Health Security Agency” (UKSHA) and the “Centers for Disease Control and Prevention” (CDC) in the US are two such examples of centralised PHS structures [5–7]. Furthermore, a diversity of organisational forms exists for conducting PHS research. In 1994, the “Essential Public Health Services” (EPHS) were defined in the US. A key aspect of the EPHS is the continuous optimisation and development of PHS activities through research and evaluation, with the aim of enhancing the quality of PHS [7]. In addition, cooperative partnerships between LHA and academic institutions have been shown to promote evidence-based interventions in the US [8,9]. Analogous structures can be found in the Netherlands, exemplified by the “academic collaborative centres for public health” [10].

The utilisation of the term “Evidence-Based Public Health” (EBPH) is on the rise, with the objective of facilitating decision-making processes grounded in scientific evidence [11]. EBPH is an approach in which public health decisions are systematically based on the best available scientific evidence, the expertise of relevant professionals, and the values and preferences of the affected population. The following principles should be observed when dealing with the issue: systematic approach, transparency in dealing with uncertainty, integration and participation, dealing with conflicts of interest and a structured, reflective process [12]. Despite the fact that EBPH in German LHA remains limited, there has been an increase in research and the publishing of results in peer-reviewed journals, although these are concentrated in a few LHA [13]. There has also been an increase in collaborations between PHS institutions and academia in recent years, with the aim of strengthening evidence-informed decision-making and supporting the implementation of newly generated knowledge [14–16]. The global pandemic caused by the corona virus has underscored the necessity for politicians to recognise the imperative to establish a scientifically grounded structure within the PHS, with the aim of contributing to the generation of evidence [17]. The present study focused specifically on the measurable components of scientific knowledge and attitudes of the staff in LHA as relevant elements of EBPH, whereas EBPH as a broader concept also encompasses aspects such as stakeholder engagement and value integration.

The German Federal Association of Physicians in the PHS (German: Bundesverband der Ärztinnen und Ärzte des öffentlichen Gesundheitsdienstes e. V.; BVÖGD) also calls for a stronger evidence-based approach, as outlined in its “mission statement for a modern PHS”, which states that the PHS should operate on a scientific basis [18]. In this context, it is important to investigate the effectiveness of public health measures at the population level and under everyday conditions [12,19]. In response to capacity gaps exposed during the COVID-19 pandemic, Germany’s federal government and the federal states agreed on the “Pact for the PHS” (German: Pakt für den Öffentlichen Gesundheitsdienst) which is a multi-year investment programme to strengthen the PHS, particularly LHA. For 2021–2026, the pact provides federal funding (€4 billion). It provides financial resources with the aim of staff expansion, enhancing the attractiveness of the PHS, facilitating digitalisation, establishing sustainable structures and implementing international health security regulations [20,21]. The pact is accompanied by an advisory board to provide guidance on its implementation and tackles the role of science and research in a future-proof PHS [17].

In recent years, various models have been developed to combine research and practice in the PHS. These include research collaborations [22–24], trainee models [25,26] and bridge professorships [27]. Furthermore, a national network for research LHA has been established since 2022 [28] and various professional societies have been founded [29]. However, LHA in Germany do not have the legal mandate to conduct research activities [10]. At the state level, state authorities assume overarching tasks, while the Robert Koch Institute (RKI), the Federal Institute of Public Health (German: Bundesinstitut für Öffentliche Gesundheit; BIÖG; formerly Bundeszentrale für gesundheitliche Aufklärung; BZgA) and the Federal Ministry of Health provide scientific expertise and play a key role in health monitoring [4,30,31].

In LHA in Germany, the following departments can be identified with relative consistency: medical service, psychiatric service, health reporting, prevention/ health promotion/ health planning, infection protection and control, child and adolescent service, crisis management, environmental medicine, dental service, digitalisation [32]. The various domains will be addressed in the subsequent sections of this article. Some departments are involved in the operational activities of the PHS, including the practical implementation of health protection and assistance. This includes monitoring hygiene in medical facilities, combatting infectious diseases (e.g., identifying sources and providing vaccinations), conducting school entrance examinations, providing advice to citizens, including individuals with mental health conditions, preparing official medical reports (e.g., fitness for duty), reviewing hygiene regulations in businesses (e.g., restaurants) and collaborating with other agencies on environmental issues (e.g., contaminated sites). Other departments are involved in health planning, promotion and reporting, where dealing with health and social determinants for needs planning and financial resource allocation are among the central tasks. LHA are organised in a heterogeneous structure and engage in a variety of activities, exhibiting different levels of intensity in addition to the prescribed legal obligations [33]. Scientific work per se is not legally binding for LHA at the municipal level in Germany and is carried out differently in the LHA due to heterogeneous structures, legal bases (federalism) and resources.

The literature shows that the PHS is increasingly focusing on evidence-based practice. However, the application of scientific methods in their own work, not just the use of existing evidence, is not yet fully developed in the various areas and departments. For example, standardised screening instruments are used or guidelines are consulted [34,35], official evidence-based recommendations are implemented [36] and planning-related data analyses are carried out on systematically collected local data [37].

In the present study, we examined the application and knowledge of scientific methods, as well as the structures for scientific work in LHA. To distinguish between scientific methods and scientific work, we used the following working definitions: Scientific work was not confined to conventional academic research. Instead, it was understood as the methodical, transparent, and evidence-based utilisation of data to facilitate the statutory obligations of LHA. It encompassed the use of the global body of knowledge, the generation of new knowledge through research, the critical analysis and verification of existing evidence, and the maintenance of awareness regarding the current state of scholarly debate. Scientific work was also understood as a communicative process: the insights gained are disseminated through publication and must be transparent, verifiable, and usable by others. In order to ensure this, scientific practice is guided by established methodological approaches and internationally recognised quality criteria [38]. By contrast, scientific methods were defined as the generation of new knowledge through the independent and systematic collection, evaluation and analysis of data. It also encompassed the planning and implementation of data collection and the communication of results in accordance with the guidelines for safeguarding good research practice [39]. In addition, scientific work was understood to include the application of scientific methods in daily professional practice, e.g., to evaluate and document the effectiveness of routine operational activities.

### Aim of the study

The objective of this study is to examine the scientific work carried out within LHA in Germany. The primary focus of this study is to assess the self-perceived knowledge of scientific methods and their practical application across diverse departments within the LHA. Additionally, the study explores the organisational structure of scientific systems within the LHA.

## Methods

In this cross-sectional study conducted in Germany, LHA staff was interviewed via an online survey. The reporting guideline improving the quality of web surveys the “Checklist for Reporting Results of Internet E-Surveys” (CHERRIES) was considered in the preparation of the article [40]. This study was advised by the Ethics Committee at the Medical Faculty of the Eberhard Karls University and the University Hospital of Tübingen. Written informed consent was implemented for all participants.

### Study design and setting

From 25 April 2024–27 June 2024, an anonymous, partially standardised online survey was conducted as part of a cross-sectional study using the “Unipark” survey software programme (Tivian XI GmbH). The target population for this survey consisted of staff of LHA in Germany. Participation in the survey was voluntary. The questionnaire was preceded by an exploratory interview study conducted in 2022, the results of which were incorporated into the development of the questionnaire [41]. The survey was initiated from a centre situated within a university hospital in Germany, the Centre for Public Health and Health Service Research (German: Zentrum für öffentliches Gesundheitswesen und Versorgungsforschung; ZÖGV).

### Survey instrument

The partially standardised questionnaire was developed in an iterative process over a period of approximately six months by an interdisciplinary team of individuals who are primarily engaged in university research or in the PHS. After structuring the themes questions were formulated, collected, sorted and subsequently reduced. The interdisciplinary development team comprised individuals with a strong background in medicine, expertise in PHS, experience in managerial roles, and substantial scientific knowledge.

In the final selection of questions, the interdisciplinary development team ensured that the questionnaire was sufficiently concise to avoid redundant questions, that the wording did not address similar topics, and that all necessary elements were covered. Filter questions were also implemented with the objective of ensuring that the questionnaire remained within an appropriate timeframe and to reduce the complexity of the questionnaire.

The team lead consistently took a proactive role, assuming leadership and taking the initiative in decision-making processes. When consensus could not be achieved within the development team, decisions were reached through internal deliberation. The ZÖGV team was responsible for selecting and prioritising the questionnaire items, scheduling meetings, and making the final decisions for the questionnaire. Along the way, the team was involved in several rounds of collaborative, participatory development of a qualitative questionnaire and feedbacking the developed items. The framework that was applied in the implementation was a participatory development method, in which PHS and scientific perspectives were incorporated. No other theoretical framework for the development of the questionnaire was conducted.

The questionnaire comprised the following topics and number of questions:

-Sociodemographics (11 questions)
-Attitude towards scientific work in the PHS (5 questions incl. 4 VAS (visual analog scale) questions)
-Respondents’ knowledge of scientific methods (13 questions incl. 2 VAS questions)
-(Research) structures in LHA (7 questions)
-Participation in research projects (network, cooperation) (1 question)

The questionnaire comprised a total of 37 items. Five of these items were filter questions. The questionnaire also incorporated six rating questions. For all other questions, except six rating questions, participants were able to select “no response” or “not applicable”. The rating questions were presented as a VAS ranging from 0 to 100 to enable finer gradations, with the numerical values not displayed to avoid monotonous response behaviour. Most questions were formulated as semi-open-ended, allowing for responses that were not considered during the development of the questionnaire. Participants were able to adjust their answers using a “Back” button. The questionnaire was made publicly accessible online via a link. The questionnaire is attached in Supplement 1. This article focuses on the self-assessed knowledge, the own application of scientific methods in comparison to the departments and positions as well as the structural conditions. Remaining results will be published in a separate article.

### Survey administration

Written informed consent was implemented for all participants. On the first page of the questionnaire, participants were informed about data protection, the aim of the study and the approximate duration of the survey. Contact information of the initiating centre and of the responsible data protection officer at Tübingen University Hospital were provided on the first page. Before the questionnaire could be initiated, the field that would be used to agree to the data processing, storage, and publication of the anonymous data had to be selected. Participants were then able to either proceed with the survey or decline participation, with no disadvantages for declining. In accordance with European General Data Protection Regulations, the use of cookies and the retention of IP addresses were prohibited, thus ensuring the integrity of the survey process and preventing any form of interruption. Data collection was anonymous, so it would not allow conclusions about individual persons. To encourage participation, three shopping vouchers were raffled off among all participants.

A pretest was conducted with a sample of 13 individuals from the development team’s network. After the pretest phase, the wording of the questionnaire was modified and the questionnaire itself was shortened.

### Recruitment

The survey was initiated on 25 April 2024 as a component of a specialist congress. On 14 May 2024, all LHA in Germany were contacted via email using their respective functional addresses or, in some cases, via contact forms. Concurrently, indirect contact was made via political bodies, state authorities and various newsletters. The recipients were requested to forward the survey to the staff in LHA. The questionnaire was to be completed by staff on an individual basis. On 11 June 2024, all LHA in Germany were contacted directly by email once more. The survey was open for a period of nine weeks.

The sample was formed using convenience sampling. Case number planning was not conducted because, at the time of study planning in early 2024, the most recent available data was from 2020. Given the substantial investments and funding in the PHS following the adoption of the Pact for the PHS, which was agreed on in 2020, the data was considered insufficiently current and therefore not suitable for the sample size planning purposes of our study.

### Data analysis

Following the conclusion of the survey, the data was exported for analysis using the IBM SPSS Statistics 28 software. This analysis was descriptive-statistical, with absolute and relative frequencies being utilised. The analysis incorporated all participants who indicated that they were working in LHA. The creation of cross tables was undertaken to facilitate the presentation of the individual groups in a clear and concise manner. The results of the rating questions were reported using the median and interquartile range (IQR), as most of these data were found to be non-normal. The analysis did not always consider the entire study population, as it was possible to refuse to answer, except for the six rating questions, and most of the questions were not designed as mandatory. Consequently, incomplete questionnaires were also included in the analysis. In addition, individual items were summarised (see Table 1).

**Table 1:** Data summary presentation.

Original items	➔	Summarized items
*(1)*Self-assessed knowledge of scientific methods (*metric scaled: 0–100)* *(2)*Own application of scientific methods *(metric scaled: 0–100)*		
Design and implementation of scientific studies (incl. organization and survey)	**➔**	*unchanged*
Systematic literature review	**➔**	Research methods
Collection of empirical data (e.g., questionnaires, laboratory values, interviews)		
Quantitative analyses (e.g., evaluation of questionnaires, laboratory values)		
Qualitative analyses (e.g., interviews, participant observation)		
Publication of papers in scientific journals	**➔**	Report
Presentation of results at scientific congresses/ specialist meetings/ conferences		
Project organization/research management (including study protocol, ethics application, study registration, data protection)	**➔**	*unchanged*
Acquisition of third-party funding	**➔**	*unchanged*
Software *(categorical)*		
SPSS	**➔**	Statistics Software
R		
Jmp		
STATA		
SAS		
Citavi	**➔**	Reference management programs
Endnote		
Zotero		
MAXQDA	**➔**	Software for qualitative data analysis
Atlas.TI		
Not applicable	**➔**	*unchanged*

### Sampling

A total of 708 individuals initiated the online questionnaire, of whom 370 completed the survey in its entirety. Assuming a total number of 26,655 people working 2024 in LHA in Germany, 2.66% of the total staff in LHA started and 1.39% completed the questionnaire [42]. The highest rate of dropout was observed in topic block 2, which focused on “perspectives on scientific work”. The average completion time was 17:05 minutes.

Table 2 presents a detailed overview of the study population stratified by position. The participants were asked to specify their place of employment. The management level was defined as health authority management, or department management. By contrast, the employees were defined as the operative level. The decision was taken not to make a distinction between “head of office” and “head of department” in order to preserve anonymity. More than a third of respondents indicated a management position (n = 194, 34.0%). The majority of participants were female. The largest proportion of respondents were between 41 and 50 years old.

**Table 2:** Characteristics of the participants categorised by position (management/ operative level) in LHA.

		Management level: LHA management/ department management	Operative level/ employee	Other	No response	Total
Gender^1^	male	72	74	3	1	**150**
	female	117	275	10	3	**405**
	no response/ other	4	6	0	1	**11**
	**total**	**193**	**355**	**13**	**5**	**566**
Age^2^	under 30 years	9	68	3	0	**80**
	31–40 years	34	110	3	3	**150**
	41–50 years	57	95	5	0	**157**
	51–60 years	62	60	1	1	**124**
	over 60 years	31	20	1	0	**52**
	no response	1	3	0	1	**5**
	**total**	**194**	**356**	**13**	**5**	**568**
Professional background *(multiple answers)*	natural sciences	21	33	2	1	**57**
	social sciences	9	68	1	0	**78**
	health sciences/ public health	35	73	3	0	**111**
	medicine	124	72	4	1	**201**
	psychology	0	9	0	1	**10**
	healthcare professional	8	76	4	0	**88**
	administrative specialist	11	24	0	1	**36**
	other	25	52	2	0	**79**
	no response	0	4	0	2	**6**
	**total**	**194**	**356**	**13**	**5**	**568**
In the PHS since...	under 2 years	14	91	1	0	**106**
	2–4 years	41	138	5	1	**185**
	5–10 years	61	82	5	2	**150**
	over 10 years	78	43	2	0	**123**
	no response	0	0	0	2	**2**
	**total**	**194**	**354**	**13**	**5**	**566**
Department *(multiple answers)*	medical services	56	36	2	0	**94**
	psychiatric service	22	24	1	0	**47**
	health reporting	35	39	3	1	**78**
	prevention/ health promotion/ health planning	51	121	4	1	**177**
	infection prevention and control	74	111	6	1	**192**
	child and adolescent health services	36	51	0	1	**88**
	crisis management	37	8	0	0	**45**
	environmental medicine	39	20	1	0	**60**
	dental service	8	5	0	0	**13**
	digitalization	36	28	1	0	**65**
	other	50	61	4	1	**116**
	no response	11	4	1	1	**17**
	**total**	**194**	**358**	**13**	**5**	**570**

1 *For anonymity: “diverse” → “no response/other”*

2 *For anonymity: “under 20” → “under 30”; “20–30 years” recoded as “under 30”*

Approximately one third of respondents had a background in medicine (n = 201, 35.4%). In total 50.9% reported a medical/healthcare background (n = 289), including 15.5% healthcare professionals (n = 88). The next most common background was in different scientific degrees: (public) health sciences (n = 111, 19.5%), social sciences (n = 78, 13.7%), natural sciences (n = 57, 10%). Fewer administrative specialists (n = 36, 6.5%) and psychologists (n = 10, 1.8%) took part in the questionnaire.

The remaining specialist backgrounds are represented by a higher number of employees than people in management positions.

## Results

### Knowledge and own application of scientific methods

**Fig 1:**
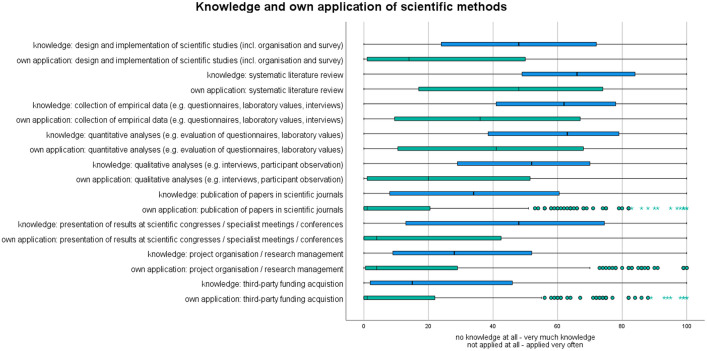
Boxplots: Knowledge and own application of ‌‌scientific methods. Boxplots display the median (MD), interquartile range (IQR), outliers (°) and extreme values (*) as well as 1.5 times the IQR (whisker)) for self-assessed knowledge and frequency of application of scientific methods. The data are based on metric scales from 0 to 100.

The self-assessment of existing knowledge of scientific methods, measured on a scale from 0 to 100, has been found to be higher overall than the information on the frequency of own application of scientific methods (see Fig 1 and Table 3).

**Table 3:** Presentation of the numerical comparisons of self-assessed knowledge and frequency of own application of scientific methods with number n, median (MD) and interquartile range (IQR).

Items of self-assessed knowledge and frequency of application	Knowledge		Own application	
*n*	*MD [IQR]*	*n*	*MD [IQR]*
design and implementation of scientific studies (incl. organization and survey)	358	47.0 [21.0-72.0]	355	10.0 [1.0-49.0]
systematic literature review	358	64.0 [46.0-83.0]	352	45.0 [15.0-72.0]
collection of empirical data (e.g., questionnaires, laboratory values, interviews)	359	60.0 [38.0-78.0]	353	33.0 [5.5-64.5]
quantitative analyses (e.g., evaluation of questionnaires, laboratory values)	360	61.0 [35.0-78.0]	351	32.0 [4.0-65.0]
qualitative analyses (e.g., interviews, participant observation)	359	50.0 [27.0-69.0]	353	18.0 [1.0-51.0]
publication of papers in scientific journals	359	30.0 [6.0-60.0]	347	1.0 [0.0-20.0]
presentation of results at scientific congresses/ specialist meetings/ conferences	359	44.0 [9.0-73.0]	349	3.0 [0.0-41.0]
project organization/research management (including study protocol, ethics application, study registration, data protection)	359	26.0 [8.0-52.0]	352	3.0 [0.0-25.0]
acquisition of third-party funding	355	14.0 [1.0-45.0]	348	1.5 [0.0-22.0]

A comparison of the departments within LHA reveals that individuals from the dental service (n = 13) consistently demonstrated the highest levels of self-assessed knowledge. Individuals from the field of health reporting (n = 78) consistently ranked second in terms of self-assessed knowledge. In terms of frequency of own application, it is noteworthy that individuals from the field of health reporting (n = 78) consistently reported the highest frequency of application, apart from the area of third-party funding acquisition. In this domain, individuals from the field of health promotion/prevention/health planning (n = 177) indicated the highest frequency of own application (see Table 4).

**Table 4:** Summarized self-assessed knowledge and own application of scientific methods in comparison with departments (multiple answers, metric scale 0–100).

*Items*		Design and implementation of scientific studies *(Item unchanged)* ^*1*^		Research methods *(Items summarized: literature review, survey, quant. and qual. analyses)* ^*1*^		Report *(Items summarized: publication, presentation at congresses etc.)* ^*1*^		Project organization/ research management *(Item unchanged)* ^*1*^		Acquisition of third-party funding *(Item unchanged)* ^*1*^	
		Knowledge	Own application	Knowledge	Own application	Knowledge	Own application	Knowledge	Own application	Knowledge	Own application
*Departments*	*n*	*MD [IQR]*	*MD [IQR]*	*MD [IQR]*	*MD [IQR]*	*MD [IQR]*	*MD [IQR]*	*MD [IQR]*	*MD [IQR]*	*MD [IQR]*	*MD [IQR]*
medical service	94	35.5 [11.5-54.0]	2.0 [0.0-30.5]	46.3 [33.4-57.8]	23.5 [6.0-42.6]	32.3 [9.0-64.0]	1.0 [0.5-19.0]	15.0 [2.0-39.0]	1.0 [0.0-10.0]	5.5 [0.0-29.5]	1.0 [0.0-14.0]
psychiatric service	47	52.0 [31.0-77.0]	9.0 [1.0-50.5]	54.6 [41.1-86.4]	41.8 [21.1-56.5]	40.8 [14.8-63.0]	2.3 [0.5-27.5]	26.0 [10.0-55.0]	8.5 [0.5-27.5]	20.0 [1.5-49.0]	1.5 [0.0-23.0]
health reporting	78	65.0 [43.0-82.0]	41.0 [3.0-62.0]	69.5 [55.0-80.5]	47.0 [26.5-60.8]	51.5 [25.0-82.0]	13.0 [2.0-50.0]	47.0 [16.0-79.0]	10.0 [0.0-52.0]	33.0 [8.0-55.0]	6.5 [0.0-34.0]
prevention/ health promotion/ health planning	177	53.5 [32.0-77.0]	31.5 [3.0-57.0]	67.1 [49.1-78.8]	47.3 [19.9-62.4]	44.3 [19.3-71.3]	6.5 [1.0-38.0]	34.5 [11.5-62.5]	9.0 [1.0-47.0]	37.5 [9.5-59.0]	12.5 [1.0-48.0]
infection prevention and control	192	47.0 [17.5-65.0]	8.0 [1.0-44.0]	56.5 [36.6-74.8]	33.0 [14.0-55.3]	29.8 [10.3-67.3]	2.5 [0.5-31.0]	24.0 [7.0-50.0]	2.0 [0.0-21.0]	9.0 [1.0-22.0]	1.0 [0.0-9.0]
child and adolescent health services	88	38.0 [10.0-58.0]	3.0 [1.0-43.0]	47.3 [32.5-65.3]	29.8 [9.0-55.8]	40.5 [8.5-65.5]	1.5 [0.5-20.0]	21.0 [3.0-49.0]	1.0 [0.0-8.0]	7.0 [1.0-22.0]	1.0 [0.0-4.0]
crisis management	45	48.5 [23.0-76.0]	11.0 [1.0-50.0]	55.5 [39.3-70.3]	31.5 [12.5-57.0]	43.5 [20.0-64.0]	4.5 [0.5-46.0]	27.5 [10.0-55.0]	3.0 [1.0-26.0]	12.5 [1.0-47.0]	1.0 [0.0-6.0]
environmental medicine	60	49.0 [23.0-77.0]	9.0 [1.0-42.0]	58.1 [42.4-75.4]	35.8 [16.5-60.8]	51.8 [20.3-78.5]	4.8 [0.5-39.5]	34.0 [12.0-57.0]	1.5 [1.0-24.0]	13.0 [1.0-47.0]	1.0 [0.0-19.0]
dental service	13	88.0 [23.0-92.0]	4.0 [3.0-79.0]	70.3 [43.0-89.5]	39.3 [35.8-85.8]	75.0 [13.0-98.0]	9.0 [1.0-91.0]	79.0 [12.0-95.0]	4.0 [1.0-91.0]	47.0 [15.0-99.0]	5.0 [0.0-88.0]
digitalization	66	47.0 [24.0-76.0]	11.0 [1.0-50.0]	57.8 [32.5-77.8]	29.3 [10.0-57.0]	44.0 [20.0-67.0]	2.0 [0.0-39.5]	36.0 [22.0-55.0]	8.0 [1.0-31.0]	20.0 [1.0-47.0]	2.0 [0.0-24.0]
other	120	46.5 [22.0-72.0]	7.5 [1.0-42.0]	54.4 [38.3-68.9]	31.8 [12.5-53.5]	31.0 [7.0-59.5]	2.0 [0.5-24.0]	23.5 [3.0-54.5]	4.0 [1.0-17.0]	12.0 [1.0-50.0]	2.0 [1.0-21.0]

^***1***^ *Presentation of the summary of the items in* Table 1

The analysis presented in Table 5 offers a comparison of self-assessed knowledge with the frequency of the own application, stratified by position (management level and employees). The findings indicate that management personnel tend to assign higher ratings to their own knowledge and frequency of application of scientific methods in domains such as design and implementation of scientific studies, report, project organisation/ research management and acquisition of third-party funding when compared with employees in these fields. However, in the domain research methods it has been observed that employees tend to self-assess their knowledge and the own application of scientific methods, roughly similar than assessed by the management level.

**Table 5:** Summarized self-assessed knowledge and own application of scientific methods in comparison with positions (single answer, metric scale 0–100).

*Items*		Design and implementation of scientific studies *(Item unchanged)* ^*1*^		Research methods *(Items summarized: literature review, survey, quant. And qual. Analyses)* ^*1*^		Report *(Items summarized: publication, presentation at congresses etc.)* ^*1*^		Project organization/ research management *(Item unchanged)* ^*1*^		Acquisition of third-party funding *(Item unchanged)* ^*1*^	
		Knowledge	Own application	Knowledge	Own application	Knowledge	Own application	Knowledge	Own application	Knowledge	Own application
*Position*	*n*	*MD [IQR]*	*MD [IQR]*	*MD [IQR]*	*MD [IQR]*	*MD [IQR]*	*MD [IQR]*	*MD [IQR]*	*MD [IQR]*	*MD [IQR]*	*MD [IQR]*
management level	194	50 [27-75]	22.5 [1.5-51]	57.25 [41-74]	33.5 [16.67-56]	51 [24-72]	12.5 [1-40.5]	36 [14-61]	5 [1-31]	20 [5-51]	2 [0-23]
operative level (employee)	358	42 [19-65]	6 [1-44]	57.5 [39.75-74.75]	35.75 [13.25-56]	25.5 [5.5-56.5]	1.5 [0.5-21]	21.5 [5-45]	2 [0-22]	12 [1-41]	2 [0-22]
other	13	40 [13-57]	13 [0-64]	71.75 [31-74.75]	54 [12.33-60.5]	27.5 [8.5-71]	1 [0-72.5]	6 [1-55]	0 [0-52]	1 [0-37]	1 [0-6]
no response	5	71 [48-94]	68.5 [64-73]	81 [76.75-85.25]	70.87 [60.5-81.25]	87 [80-94]	77.75 [76.5-79]	71.5 [48-95]	68.5 [55-82]	49.5 [11-88]	42 [0-84]

^***1***^ *Presentation of the summary of the items in* Table 1.

In the present study, 75.7% (n = 277) of respondents indicated that they had attended scientific conferences in the past two years without making a personal contribution. Conversely, 21.4% (n = 89) of respondents reported presenting their own research results at scientific conferences. Furthermore, 73.4% (n = 262) of respondents indicated that they had not participated in publications in scientific journals (peer-reviewed) in the last five years. However, 24% (n = 86) of respondents indicated that they had been involved in publications. This data reveals that 81.9% of the employees (n = 176) have not contributed to publications, in comparison with 60.6% of managerial staff (n = 77).

The results indicate that 76.5% (n = 270) of respondents reported a need for scientific literature in their daily work, alongside 73.5% (n = 261) of respondents admitting to reading scientific journals. The journals most frequently read by the respondents were primarily national in scope, including national journals like “Das Gesundheitswesen”, “Ärzteblatt” and “Bundesgesundheitsblatt”. In contrast, international journals were mentioned by fewer respondents (Lancet, NEJM, BMJ). Furthermore, a significant proportion of respondents, specifically 87.2% of managers (n = 109) and 64.7% of employees (n = 139), acknowledged that reading scientific journals is an integral part of their daily professional routine. The highest proportions of respondents reporting the use of scientific journals were found among those working in infection control (36.4%, n = 95) and in prevention/ health promotion/ health planning (34.5%, n = 90). In contrast, the lowest proportions were observed among those employed in dental services (2.7%, n = 7) and psychiatric services (8.0%, n = 21). Furthermore, the findings reveal that 11.5% (n = 41) of participants had experience as a reviewer for scientific journals.

The results indicate that 30.1% (n = 105) of respondents reported the presence of management staff in their LHA who prepare applications for project funding, while 24.6% (n = 86) reported management staff who publish in scientific journals. The items “There are people in leadership positions... who (1) write applications for project funding; (2) publish in scientific journals” in comparison with the use of research methods (summarized item) show that people who state that they have scientifically active persons in leadership positions in their LHA use scientific methods more often (45.0 [18.5–62.5], n = 137) than those reporting an absence of scientifically active leaders (30.8 [12.0–52.0], n = 216).

### Structural conditions

In this study, 60.2% (n = 213) of respondents stated that they did not have access to or used any of the software presented in the questionnaire to support scientific work, whilst 15.5% (n = 55) indicated that they had access to and actively employed the software SPSS and 8.8% (n = 31) reported using the software R. The following distribution was observed when the surveyed software products were grouped: 26.6% (n = 94) own and use statistical software, 10.7% (n = 38) own and use citation and reference management programmes and 2% (n = 7) own and use software for qualitative data analysis.

The majority (71.7%, n = 256) indicated a need for further training/counselling on scientific methods. The preference for further training was expressed by 91.4% (n = 234) of respondents, while 59.4% (n = 152) indicated a preference for counselling. Employees were more likely to express a need for training/counselling on scientific methods (74.5%, n = 161) than managers (67.5%, n = 85).

## Discussion

The results of the SCOPE questionnaire demonstrate the potential for the implementation of scientific work in LHA in Germany. The high self-assessed knowledge in all scientific methods combined with a low frequency of application may indicate existing structural, organisational, and legal barriers to research activities – knowing that in the past, research was not part of the statutory mandates of LHA.

### Knowledge and own application of scientific methods in LHA

Interpreting both the frequency of use and the self-assessed competencies across scientific methods suggest a clear gradient among staff working in LHAs: respondents reported a higher level of knowledge and frequency of use of comparatively common methods, (e.g., quantitative analyses of questionnaires or laboratory values), whereas more advanced activities (e.g., study design development or third-party funding acquisition) were associated with a lower level of knowledge and frequency of use.

National scientific literature is used more frequently by staff at LHA in Germany than international literature. International journals may have been used less frequently to date due to limited access or funding opportunities. At the same time, the focus on national publications may reflect the responsibilities and structure of the PHS. Since 2024, access to literature for the PHS has been established through the Academy for Public Health (German: Akademie für Öffentliches Gesundheitswesen; AÖGW), which should enable broader use of international scientific sources in the future [43].

The survey results also show that employees have so far been barely involved in scientific publications. In contrast to scientific institutions, LHA often have fewer incentives to publish findings, as this is not explicitly part of the institutions’ tasks or objectives. To date, there is a lack of support for scientific work in the PHS, particularly in the form of further development or career opportunities and incentives for employees [41,44].

### Structural conditions for scientific work in LHA

The results show that research infrastructure (e.g., software) is only available to a limited extent of staff working in LHA. Without appropriate tools and structural prerequisites, scientific work in the daily routines of the PHS is hardly feasible. The Pact of the PHS provides, among other things, funding for the expansion of infrastructure and digitalisation, which could also benefit scientific work [20,21].

The rare application of scientific methods may also indicate a possible lack of experience with the topic or a lack of methodological competence. Low-threshold, practice-oriented training courses on scientific methods, such as those offered by the ZÖGV [25,45] or AÖGW [46], can be a suitable measure to promote interest and skills in the field of scientific work. The high demand for continuing education and consulting services demonstrates that there is fundamental motivation and potential for applying scientific methods. At the same time, it suggests that existing offerings are not sufficiently tailored to the needs of the target group or are unknown, as otherwise the need would not be so pronounced. There may be a need for optimisation in terms of content, formats, public relations activities, or even staff time and financial resources in PHS. Additional elements such as trainee programmes at various institutions enable public health staff to gain insights, cooperate directly with research institutions, and improve their methodological skills through practical experience [47–49]. As these approaches are still relatively new, systematic evidence on their effectiveness is not yet available. Collaborations between PHS and university or scientifically oriented institutions, as well as bridge professorships, are another approach that could increase scientific work at LHA [22–24,27,48,50]. To increase knowledge of scientific methods in the PHS, opportunities for student scientific qualification (e.g., bachelor’s and master’s theses or clinical internships and dissertations) in the PHS would be necessary [51].

The literature emphasizes the need to systematically embed scientific work in the various departments of LHA. The use of suitable laboratory methods is recommended for infection control. For preventive measures, scientifically sound recommendations from the fields of medicine, public health, health psychology, and sociology should be used. Furthermore, it is recommended that the formulation of expert opinions be underpinned by scientific principles to ensure the formulation of legally sound statements [51,52]. The results of the SCOPE questionnaire show that the use of scientific methods varies greatly between departments. Regarding self-assessment of one’s own knowledge and the frequency of applying scientific methods, particularly high scores were recorded in the departments of health reporting and prevention/health promotion, especially in the areas of scientific methods and reporting. In contrast, lower scores were observed in the department of infection control, primarily in scientific methods and project organisation. This discrepancy suggests that the requirements formulated in the literature have so far only been partially implemented in practice. In addition, the establishment of scientific work depends heavily on the structural and professional framework conditions of the respective departments.

A potential barrier is the absence of a legal mandate for scientific work in most federal state public health laws in Germany, leaving it secondary to core tasks such as protection, prevention, and surveillance [41]. This may also explain respondents’ low assessment of applying their skills.

In practice, this structural prioritisation is reflected in daily workload and capacity constraints within LHA. Most staff resources within LHA are already preoccupied with mandatory, required, routine tasks. The implementation of non-mandatory activities is often challenging [41]. The introduction of additional tasks such as scientific work is even more challenging, as it also entails the coordination and integration of new activities without corresponding relief or additional resources.

The ongoing integration of externally third-party funded projects within the PHS has the potential to enhance scientific work by establishing stable structures for evidence-based work, critical appraisal, and transparent outcome-dissemination. Furthermore, it can encourage the systematic application of scientific methods in routine operational practice [48,53–55]. However, third-party funded projects alone might not be sufficient to ensure lasting structural integration, as durable anchoring requires additional institutional frameworks, protected resources, and long-term capacities beyond the time-limited funding period.

### Workforce and Leadership for Scientific Work in LHA

One result demonstrates the influence of managers on employees’ scientific work. Employees are more likely to engage in scientific methods if their managers are also scientifically active. Therefore, not only legal anchoring and appropriate human resources are required, but also structural prerequisites and a conscious attitude on the part of managers regarding the relevance of scientific work in the PHS. Targeted training opportunities for managers could make an important contribution to raising awareness and promoting such an attitude.

In this study, a total of 71.55% of female respondents participated, in contrast to 26.5% male respondents. Moreover, the monitoring of healthcare personnel data indicates that, in 2024, female personnel constituted 81.97% of all personnel employed within the PHS, with the remaining 18% of personnel employed within the PHS being male [42].

Limited human resources, particularly within public health professionals [56], as well as a significant decline in the number of medical professionals [57], can impair the application of scientific methods, as they significantly limit the available capacities of existing staff. The regional health personnel monitoring for the PHS, published in September 2024, does not include scientific work or the application of scientific methods among the detailed lists of areas of activity of the PHS [42], which in turn underscores the lack of a defined activity for scientific work in the PHS. Only 8.14% of staff work in the area of “coordination, communication, moderation, advocacy, policy advice, quality assurance”, which might be closest to scientific work [42].

The interdisciplinary composition of many LHA offers potential for scientific work [58]. The methodological expertise of an interdisciplinary team is broad and highly valuable. Teams with different professional backgrounds have a wide range of knowledge about survey instruments and analysis methods and can support each other in interpreting results. Nevertheless, further development of standards and structures in the area of scientific work in LHA seems sensible in order to increase quality and enable a certain degree of standardisation, as is sought through instruments such as the Public Health Action Cycle (PHAC) [59].

A comparison with other medical disciplines in Germany, e.g., with general practice (GP), shows that the now established GP research practices, supported by a “Research Ready Concept”, are increasingly participating in research in primary care. The Research Ready Concept is a quality assurance concept for practice teams wishing to participate in primary care research and includes standards for the qualification level for different types of studies (acquisition of knowledge and skills) [60,61]. Concurrently, an increase has been observed in scientific activities, encompassing the integration of research into the curriculum in the study of human medicine [62], thus strengthening competencies in this area.

Among the participants, there were significantly more people with a medical background than members of other professions, which may have distorted the results. The monitoring of healthcare personnel indicates that, in 2024, 20% of medical professionals were employed within the PHS [42]. In contrast, in this study, roughly 35% of the participants had a medical background, suggesting a potential discrepancy between the observed and expected percentages. One possible reason for the increased participation of people with a medical background could be recruitment via functional email addresses of the LHA. It is possible that the invitation to participate in the questionnaire was not forwarded to all employees, but only to a specific group of people, for example, those with a medical background and management position. The distribution of respondents shows that 62.8% work in operational positions and 34% in managerial positions. This distribution does not reflect the actual situation in LHA, as a significantly higher proportion of people working in operational positions can be expected.

The highest dropout rate among participants was recorded in the “Attitudes toward academic work” section. Asking opinions, perspectives, and attitudes toward academic work at the beginning of the questionnaire (after demographics) may have had a deterrent effect, especially among individuals who previously had little or no connection to academic work. It is also likely that the dropout at this point led to an exacerbated selection bias, with primarily individuals with an interest in academic work continuing to participate in the survey.

### Strengths and limitations

Due to the use of convenience sampling, it is not possible to generalise the results. The occasionally low participation rates should be interpreted with caution, as they may indicate potential selection bias. During the design phase, the use of validated survey instruments such as the Munich Research Competence Scale [63] was considered but rejected due to the unsuitability of their content orientation. It can be assumed that the survey reached people from the PHS who are interested in scientific work. Due to the questionnaire’s origin from a university hospital, it can be assumed that response behaviour could be influenced by social desirability. Social desirability, in the context of survey research, is defined as the propensity of respondents to respond in a manner that aligns with the expectations of their peers, thereby potentially distorting their actual thoughts and behaviours [64]. This bias has the potential to distort data, particularly in surveys addressing sensitive or socially charged topics [64]. To address the issue of social desirability, an anonymous survey method was selected. In some cases, indirect questions were employed (e.g., the question regarding the own application of publications and the question about the frequency of publications). A scale was selected using the 0–100 scale, which ensures that it is not possible to always select the same value. The sample size was not possible to be planned in advance, since the LHA structures in Germany are heterogeneous, with different designations for work and administrative areas. A high number of people selected “other” when asked about their area of activity supports this thesis. It was not possible to analyse the data by LHA due to anonymity.

The interdisciplinary design of the survey instrument, which considers different perspectives and experiences, is a key strength of the survey. To the best of our knowledge, this survey is the first of its kind to be conducted at a national level in Germany on this topic. The absence of mandatory questions in the survey design may have had a positive effect on the drop-out rate, and the option to select “other” or “no response” further emphasises this. The maintenance of survey participant anonymity, the option for voluntary participation, and the provision of an opportunity to participate in a competition have also been identified as contributing factors to the heightened willingness to partake in the survey. It is recommended that the survey instrument undergo replication in the future, with the possibility of subsequent development, to facilitate comparative analysis.

When interpreting the results regarding frequency of use of scientific methods, it should be noted that the question about frequency of use was asked within the last five years and that respondents who have been working in the PHS for less than five years also participated in the questionnaire (292 of 569; 51.32%). However, since these respondents may also have used the surveyed methods as part of their work in the PHS, they were not extracted. A substantial proportion of respondents selected “medicine” as their professional background (n = 201, 35.4%), suggesting potential bias due to the overrepresentation of physicians.

## Conclusion

The results demonstrate that according to LHA staff there is potential and interest in establishing scientific practice in PHS. This establishment requires a complex interplay of various prerequisites, including legal anchoring, structural support, sufficient human resources, needs-based continuing education opportunities, and a leadership culture that specifically promotes scientific work and the use of scientific methods.

Both a fundamental interest in and existing basic scientific method competencies are present in the PHS. The discrepancy between the high self-assessed knowledge of scientific methods and its low frequency of application in everyday life could indicate a structural deficit. Scientific work or the use of scientific methods may thus fail less due to a lack of motivation than due to a lack of organisational and legal prerequisites.

For the sustainable promotion of scientific work in LHA, targeted investments in infrastructure, training, and institutional frameworks are necessary. Equally important is a clear positioning of scientific work within the organization so that it is perceived as an integral part of the responsibilities of LHA and does not remain behind mandatory, required, routine tasks such as infection control and surveillance.

## Supporting information

S1 File SCOPE Questionnaire (original). (PDF)
