# Supplementary material for: Scientific work and knowledge of scientific methods in local public health authorities (SCOPE) – wish or reality? Results of a semi-standardized cross-sectional survey of local public health authorities in Germany (Part 1)
Source: PLoS One. 2026 Apr 9;21(4):e0345944. doi: 10.1371/journal.pone.0345944 (PMC13065011; doi:10.1371/journal.pone.0345944)
Supplement: S1 File — (PDF) [file pone.0345944.s001.pdf]

SCOPE Questionnaire - **Sc**ientific work and knowledge **o**f scientific methods in local **p**ublic h**e**alth authorities (LHA) in Germany

This eSupplement contains an English translation of the original German questionnaire. It does not represent the original questionnaire.

Dear Colleagues,

Research in the public health service (PHS) is a hot topic, but little is known about the current state of affairs in Germany. A collaborative effort involving various local health authorities (LHA) and a university institution has been launched to investigate this issue. With this questionnaire, we would like to invite you to provide a nationwide overview of how you apply scientific methods in your daily work, as well as your attitudes and knowledge on the subject. We would like to learn about your perspective and your experiences within the LHA.

If you hold roles at multiple levels within the PHS (e.g., at the state and local levels), please refer to your role within the LHA when answering the questionnaire.

Participation in this survey is voluntary and takes 15–20 minutes. You can stop participating at any time without any negative consequences by simply closing the survey window. The survey is anonymous, meaning no personal data will be collected or processed as part of the study. You cannot pause the questionnaire once you start.

If you complete the entire questionnaire, you'll have the chance to win one of five "Wunschgutscheinen" worth €10. To enter, simply indicate at the end of the survey that you'd like to participate in the drawing. You'll then be redirected to a separate page. Your email address cannot be linked to your responses.

Do you have any questions about the study? Please feel free to contact us:

Zentrum für öffentliches Gesundheitswesen und Versorgungsforschung
Universitätsklinikum Tübingen
Osianderstr. 5, 72076 Tübingen
Tel.: 07071 29-87427
Email: zoegv@med.uni-tuebingen.de

Thank you very much for taking the time to complete this questionnaire and for helping us gain a clear picture of the current state of scientific work in the PHS in Germany!

Contact the Data Protection Officer:

Universitätsklinikum Tübingen, Data Protection Officer Sven Sender, Geissweg 3, 72076 Tübingen, Telefon: 07071 29-87667, E-Mail: dsb@med.uni-tuebingen.de

For more information, please click here

- ○ By participating in this survey, I consent to the processing and storage of my data, as well as the publication of anonymous data.
- ○ I do not wish to participate.

START SURVEY

ENGLISH TRANSLATION OF THE QUESTIONNAIRE

SCOPE Questionnaire - **Sc**ientific work and knowledge **o**f scientific methods in local **p**ublic h**e**alth authorities (LHA) in Germany

Demographics

Demographic information is collected in order to examine differences in response patterns among the various groups.

1. In which federal state do you work in the public health service (PHS)?

- ○ No response
- ○ Baden-Württemberg
- ○ Bavaria
- ○ Berlin
- ○ Brandenburg
- ○ Bremen
- ○ Hamburg
- ○ Hesse
- ○ Mecklenburg-Western Pomerania
- ○ Lower Saxony
- ○ North Rhine-Westphalia
- ○ Rhineland-Palatinate
- ○ Saarland
- ○ Saxony
- ○ Saxony-Anhalt
- ○ Schleswig-Holstein
- ○ Thuringia

2. Which health authority do you work for?

- ○ Local health authority (e.g., public health department)
- ○ State-level health authority (e.g., state health department or state office)
- ○ Federal health authority (e.g., ministry or specialized agency)
- ○ Federal agency (e.g., RKI, BMG)
- ○ Other: _____
- ○ No response

3. In which department or division do you work? *Multiple answers allowed.*

Please select the department that best applies to you.

- ○ Medical Service
- ○ Psychiatric Service
- ○ Health Reporting
- ○ Prevention/ Health Promotion/ Health Planning
- ○ Infection Prevention And Control
- ○ Child and Adolescent Health Services
- ○ Crisis Management
- ○ Environmental Medicine
- ○ Dental Services
- ○ Digitalization

ENGLISH TRANSLATION OF THE QUESTIONNAIRE

- ◯ Other: _____
- ◯ No response

4. What is your job title?

- ◯ Executive level: LHA director or head of department
- ◯ Staff/operational level
- ◯ Other: _____
- ◯ No response

5. How many people live in the jurisdiction of the LHA where you work?

- ◯ up to 100,000 people
- ◯ 100,001–200,000 people
- ◯ 200,001–300,000 people
- ◯ 300,001–400,000 people
- ◯ 400,001–500,000 people
- ◯ 500,001–1,000,000 people
- ◯ over 1,000,000 people
- ◯ I don't know
- ◯ No response

6. How many years have you been working in the PHS?

- ◯ Less than 2 years
- ◯ 2–4 years
- ◯ 5–10 years
- ◯ More than 10 years
- ◯ No response

7. What is your current workweek at the LHA (in hours per week)?

- ◯ 39
- ◯ Not specified
- ◯ 41
- ◯ 30
- ◯ 40
- ◯ 20
- ◯ 39.5
- ◯ 35
- ◯ 32
- ◯ Enter number of hours per week: _____

8. What is your academic background? *Multiple answers allowed*

- ◯ Natural sciences

ENGLISH TRANSLATION OF THE QUESTIONNAIRE

- ◯ Social sciences
- ◯ Health sciences / Public health
- ◯ Medicine
- ◯ Psychology
- ◯ Healthcare professional
- ◯ Administrative specialist
- ◯ Other: _____
- ◯ No response

9. Please indicate your highest level of education.

- ◯ Secondary school diploma / Intermediate school diploma / High school diploma
- ◯ Vocational training
- ◯ Bachelor's degree: University of Applied Sciences
- ◯ Bachelor's degree: University
- ◯ Master's degree / Diploma: University of Applied Sciences
- ◯ Master's degree / Diploma: University
- ◯ Medical license
- ◯ Doctorate: Dr. med. / Dr. med. dent.
- ◯ Doctorate: other, e.g., Dr. rer. nat., PhD
- ◯ Habilitation
- ◯ Other: _____
- ◯ No response

10. What is your gender?

- ◯ Male
- ◯ Female
- ◯ Diverse
- ◯ No response

11. How old are you?

- ◯ Under 20
- ◯ 20–30 years old
- ◯ 31–40 years old
- ◯ 41–50 years old
- ◯ 51–60 years old
- ◯ Over 60 years old
- ◯ No response

ENGLISH TRANSLATION OF THE QUESTIONNAIRE

Perspectives on Scientific Work in PHS

Below, we present questions regarding your perceptions, attitudes, and understanding of scientific work in PHS.

12. In the following, we would like to learn more about your views on scientific work in LHA.

Please rate the following statements on a scale from "strongly disagree" to "strongly agree."

When applying scientific methods in LHA, it is not necessary to assess ethical aspects	strongly disagree --------------- strongly agree
LHA should have access to an independent body that provides advice and support on issues related to good scientific practice	strongly disagree --------------- strongly agree
LHA should make their collected data publicly available	strongly disagree --------------- strongly agree
LHA should provide ongoing scientific training	strongly disagree --------------- strongly agree
LHA should have the opportunity to carry out scientific projects	strongly disagree --------------- strongly agree
LHA should publish their findings primarily in scientific journals	strongly disagree --------------- strongly agree
Scientific methods should be applied in LHA	strongly disagree --------------- strongly agree

13. What factors do you consider conducive to scientific work in your day-to-day activities at the LHA?

Please rate the following statements on a scale from "strongly disagree" to "strongly agree."

Evidence generation	strongly disagree --------------- strongly agree
Increasing activity in the field of PHS	strongly disagree --------------- strongly agree
Generating new knowledge	strongly disagree --------------- strongly agree
Personal development / career progression	strongly disagree --------------- strongly agree
Promoting the further development of public health and administration in the spirit of evidence-based practice	strongly disagree --------------- strongly agree
External impact: personal (e.g., own career path)	strongly disagree --------------- strongly agree
External impact: institutional (e.g., image of the LHA)	strongly disagree --------------- strongly agree
I do not apply scientific methods in my daily work	strongly disagree --------------- strongly agree

ENGLISH TRANSLATION OF THE QUESTIONNAIRE

14. What factors do you perceive as hindering scientific work in your day-to-day duties at the LHA?

Please rate the following statements on a scale from "strongly disagree" to "strongly agree."

No research infrastructure available for scientific work (software, access to scientific databases, etc.)	strongly disagree --------------- strongly agree
No specific expertise in scientific work	strongly disagree --------------- strongly agree
Lack of commitment from management (department head/agency director/county administrator)	strongly disagree --------------- strongly agree
Lack of legal basis	strongly disagree --------------- strongly agree

Lack of resources: time, funding, personnel	strongly disagree --------------- strongly agree
No need to apply scientific methods in my day-to-day work at the LHA	strongly disagree --------------- strongly agree
There are no barriers	strongly disagree --------------- strongly agree

15. What do you consider to be part of scientific work at a LHA? *Multiple answers allowed*

- ◯ Grant applications
- ◯ General project management / organization of a research project (data protection, timelines, budget management, etc.)
- ◯ Data collection
- ◯ Data analysis (quantitative, e.g., questionnaires, lab results; qualitative, e.g., interviews)
- ◯ Data preparation in various formats (including for non-specialists)
- ◯ Publication in scientific journals and formats
- ◯ Publication in non-scientific media (e.g., newspapers)
- ◯ Development of standards and guidelines
- ◯ Development of research questions
- ◯ Structured search for findings in scientific journals
- ◯ Assessment of evidence quality and evaluation of sources
- ◯ Presentations at scientific conferences
- ◯ Teaching activities
- ◯ None of the above
- ◯ Other: _____
- ◯ No response

16. Please rate the following statements about scientific work in LHA on the scale. *The statements are taken from interviews with employees of LHA in the federal state Baden-Württemberg in Germany, conducted in the summer of 2022.*

Please rate the following statements on a scale from "strongly disagree" to "strongly agree."

"We're just a regular department in human medicine. […] So any argument in favor of research that you might hear at other institutes […] applies just as much to us."	ORIGINAL: „Wir sind ein ganz normales Fach in der Humanmedizin. […] Also jedes Argument für Forschung, was Sie jetzt an anderen Instituten haben […], zählt für uns genauso"	strongly disagree --------------- strongly agree
"I don't think every LHA has to do this on its own. I would certainly like it if we were essentially data providers who then handed everything else over to someone else, who would then essentially feed the results back to us, and we	ORIGINAL: „Wir sind ein ganz normales Fach in der Humanmedizin. […] Also jedes Argument für Forschung, was Sie jetzt an anderen Instituten haben […], zählt für uns genauso"	strongly disagree --------------- strongly agree

ENGLISH TRANSLATION OF THE QUESTIONNAIRE

could then do something with them accordingly.”		
“Well, of course we always refer to scientific reports or the research being conducted there. I suppose you always have to keep up with the literature.”	ORIGINAL: „Wir sind ein ganz normales Fach in der Humanmedizin. […] Also jedes Argument für Forschung, was Sie jetzt an anderen Instituten haben […], zählt für uns genauso“	strongly disagree --------------- strongly agree

ENGLISH TRANSLATION OF THE QUESTIONNAIRE

Knowledge of scientific methods

In this section, we would like to ask you about your knowledge of scientific methods and scientific work. We are also interested in learning what topics interest you and where there is a need for further support.

17. Please use the scale below to indicate how you rate your knowledge in the following areas.

Please rate the following statements on a scale from "no knowledge at all" to "a lot of knowledge" (layperson to expert)

design and implementation of scientific studies (incl. organization and survey)	no knowledge at all -------------- a lot of knowledge
systematic literature review	no knowledge at all -------------- a lot of knowledge
collection of empirical data (e.g. questionnaires, laboratory values, interviews)	no knowledge at all -------------- a lot of knowledge
quantitative analyses (e.g. evaluation of questionnaires, laboratory values)	no knowledge at all -------------- a lot of knowledge
qualitative analyses (e.g. interviews, participant observation)	no knowledge at all -------------- a lot of knowledge
publication of papers in scientific journals	no knowledge at all -------------- a lot of knowledge
presentation of results at scientific congresses/ specialist meetings / conferences	no knowledge at all -------------- a lot of knowledge
project organization/research management (including study protocol, ethics application, study registration, data protection)	no knowledge at all -------------- a lot of knowledge
acquisition of third-party funding	no knowledge at all -------------- a lot of knowledge

18. Please indicate on the scale how often you have used the following scientific methods over the past 5 years.

Please rate the following statements on a scale from "not at all" to "very often"

design and implementation of scientific studies (incl. organization and survey)	not at all ------------- very often
systematic literature review	not at all ------------- very often
collection of empirical data (e.g. questionnaires, laboratory values, interviews)	not at all ------------- very often
quantitative analyses (e.g. evaluation of questionnaires, laboratory values)	not at all ------------- very often
qualitative analyses (e.g. interviews, participant observation)	not at all ------------- very often
publication of papers in scientific journals	not at all ------------- very often
presentation of results at scientific congresses/ specialist meetings / conferences	not at all ------------- very often
project organization/research management (including study protocol, ethics application, study registration, data protection)	not at all ------------- very often
acquisition of third-party funding	not at all ------------- very often

ENGLISH TRANSLATION OF THE QUESTIONNAIRE

19. How many scientific conferences or (specialized) meetings have you attended as a participant in the past two years? (excluding those where you presented) *(Filter question / Required question)*

- ◯ 1–3
- ◯ 4–6
- ◯ 7–10
- ◯ >10
- ◯ None
- ◯ Not specified

19.1. At which scientific conferences or (specialized) meetings did you attend without presenting your own work (lecture or poster)?

- ◯ bvÖgd
- ◯ No response
- ◯ _____

20. How many scientific conferences or (specialized) meetings have you attended in the past 2 years where you presented a paper (oral presentation or poster)? *(Filter question / Required question)*

- ◯ 1–3
- ◯ 4–6
- ◯ 7–10
- ◯ >10
- ◯ None
- ◯ No response

20.1. At which scientific conferences or (specialized) meetings did you present a paper (oral presentation or poster)? *(Filter question)*

- ◯ bvÖgd
- ◯ No response
- ◯ _____

21. How have you communicated your results/findings over the past 5 years? *(Filter question / Required question), Multiple answers allowed*

- ◯ Peer-reviewed journal
- ◯ Non-peer-reviewed journal
- ◯ Journal (unknown whether peer-reviewed)
- ◯ Policy paper
- ◯ At conferences/conventions/workshops in the form of presentations/posters
- ◯ Consultation in committees
- ◯ Other forms of written summaries/reports outside of scientific journals
- ◯ Non-print media (e.g., Internet, TV, radio)
- ◯ Within the administrative unit (e.g., LHA, county office, city)
- ◯ Not at all
- ◯ Other: _____
- ◯ No response

ENGLISH TRANSLATION OF THE QUESTIONNAIRE

21.1. In which peer-reviewed journals have you published your results? *(Filter question if the journal is peer-reviewed)*

- ○ ______

22. How many peer-reviewed scientific journal articles have you authored in the past 5 years? *This may include articles published before you began working at the public health department within this 5-year period*

- ○ 1–3
- ○ 4–6
- ○ 7–10
- ○ More than 10
- ○ None
- ○ No response

23. Have you ever served as a reviewer for a peer-reviewed academic journal?

- ○ Yes
- ○ No
- ○ No response

24. How has the pandemic affected your publishing habits?

The number of publications I contribute to each year...

- ○ ...has decreased
- ○ ...has remained the same
- ○ ...has increased
- ○ No response

25. Do you have a need for training or consulting services related to scientific methods? (*Filter question / Required question)*

- ○ Yes
- ○ No
- ○ No response

25.1. What are your needs regarding training or consulting services?

- ○ Training
- ○ Consulting
- ○ Other: _____
- ○ No response

ENGLISH TRANSLATION OF THE QUESTIONNAIRE

Structures in the PHS

This section aims to identify what "research and scientific infrastructure" is available and/or in use at LHA and to what extent there are plans for its expansion.

26. Which software do you have access to and actively use at your LHA? *Multiple selections allowed*

- ○ SPSS
- ○ R
- ○ JMP
- ○ STATA
- ○ SAS
- ○ MAXQDA
- ○ Atlas.TI
- ○ Endnote
- ○ Zotero
- ○ Citavi
- ○ None of the above
- ○ Other: _____
- ○ No response

27. Do you need scientific literature for your daily work at the LHA?

- ○ Yes
- ○ No
- ○ No response

28. Do you read scientific journals as part of your daily work at the LHA? (*filter question / Required question)*

- ○ Yes
- ○ No
- ○ No response

28.1. If so, which academic journals do you read?

- ○ _____
- ○ No response

29. Does your LAH have any plans for scientific projects?

By "scientific projects," we mean projects that go beyond the department's statutory mandate.

- ○ Yes
- ○ No
- ○ I don't know

ENGLISH TRANSLATION OF THE QUESTIONNAIRE

- ◯ No response

30. At my LHA, there are people in leadership positions who… (*Multiple answers allowed)*

- ◯ ...hold a Ph.D.
- ◯ ...have completed their habilitation
- ◯ ...publish in scientific journals
- ◯ ...write grant proposals
- ◯ None of the above
- ◯ I don't know
- ◯ No answer

31. What structural requirements do you need to be able to conduct scientific work? (*Multiple answers allowed)*

- ◯ Scientific staff
- ◯ Research budget (staff costs, publication, ethics application, etc.)
- ◯ Research mandate from the district administrator or department head
- ◯ Support from higher authorities (state or federal level)
- ◯ Professional development opportunities
- ◯ Advisory services
- ◯ Internal professional networks
- ◯ Collaboration with LHA
- ◯ Partnerships with universities/colleges
- ◯ I do not need anything
- ◯ Other: _____
- ◯ I don't know
- ◯ No response

ENGLISH TRANSLATION OF THE QUESTIONNAIRE

Participation in Research Projects (Networking / Collaboration)

In the following, we would like to ask you about your participation in research projects. Here, we are referring to collaborations—that is, planned cooperation between institutions and the mutual alignment of objectives and processes. In a collaboration, cooperation is based on equality and occurs on a regular basis.

32. Please indicate which of the following institutions you have collaborated with over the past 5 years to carry out research activities. (*Multiple answers allowed)*

- ○ Local health authority (e.g., public health department)
- ○ Intermediate health authority (e.g., state health department or state office)
- ○ Higher-level health authority (e.g., ministry or specialized agency)
- ○ Federal agency (e.g., RKI, BMG)
- ○ University
- ○ (Technical) college
- ○ Independent research institute (e.g., IGES, aQua, IQTIG)
- ○ Not affiliated with any institution
- ○ Other: _____
- ○ I don’t know
- ○ No response

ENGLISH TRANSLATION OF THE QUESTIONNAIRE

End of the Survey

Do you have any additional comments?

(e.g., free associations related to the topic, things you felt were missing from the questionnaire, suggestions regarding the scientific rigor of PHS)

- ◯ ___________________

Would you like to enter the giveaway and win one of 5 gift certificates worth €10?

Enter the giveaway

We would like to thank you very much for your participation!

ENGLISH TRANSLATION OF THE QUESTIONNAIRE
